# Oral Health and Teledentistry Interest during the COVID-19 Pandemic

**DOI:** 10.3390/jcm10163532

**Published:** 2021-08-11

**Authors:** Magdalena Sycinska-Dziarnowska, Marzia Maglitto, Krzysztof Woźniak, Gianrico Spagnuolo

**Affiliations:** 1 Department of Orthodontics, Pomeranian Medical University in Szczecin, Powstańców Wielkopolskich Street 72, 70111 Szczecin, Poland; magdadziarnowska@gmail.com (M.S.-D.); krzysztof.wozniak@pum.edu.pl (K.W.); 2 Department of Neurosciences, Reproductive and Odontostomatological Sciences, University of Naples “Federico II”, 80131 Napoli, Italy; mar.maglitto@gmail.com; 3 Institute of Dentistry, I. M. Sechenov First Moscow State Medical University, 119435 Moscow, Russia

**Keywords:** oral health, teledentistry, Google Trends, COVID-19 pandemic

## Abstract

Background: The COVID-19 pandemic outbreak has significantly changed access to dental treatments. Methods: The data related to oral health and teledentistry topics were collected from the open database Google Trends. The analyzed material was collected from 19 June 2016 to 6 June 2021 among anonymous search engine users. The following expressions were analyzed: “dental care”, “emergency dental care”, “oral health”, ”periodontitis”, “teledentistry”, “is it safe to go to the dentist”, and “COVID-19” and ”PPE dentist”. Results: During the first lockdown in 2020, a significant increase in “emergency dental care” phrase queries was detected, with a simultaneous decrease in regular “dental care” questions, as well as a peak in the queries for “periodontitis” preceded by lower interest in “oral health.” The number of searches stated for “teledentistry” increased during the time of the pandemic 5 times and for and “PPE dentist” 30 times. The risk of visiting the dental studio was seen in almost 40 times increase in the query “is it safe to go to the dentist.” Conclusions: The COVID-19 imprinted a stigma on oral health care. In this difficult epidemiological situation, teledentistry might become a helpful solution.

## 1. Introduction

In late June 2021, about 182 million COVID-19 cases were reported globally, with almost 4 million people deaths [1]. It seems very likely that the COVID-19 pandemic had an impact on oral health-related behaviors. The stringent measures undertaken to limit the spread of COVID-19 disease with social distancing rules made access to dental offices more troublesome. The risk of contagion can be reduced by introducing teledentistry [2]. Teleconsultation, which begins with an online triage, was a compromise that limited patient access but also ensured effective treatment and relief from symptoms [3].

According to some studies, routine dental care and emergency dental care have been immensely influenced by the waves of COVID-19 [4,5,6,7]. The large impact of the COVID-19 pandemic on dentistry was shown in the study conducted by Soltani et al., in which as many as 659 articles published by dental journals were found in PubMed regarding implications of the COVID-19 pandemic in dentistry [8]. Moreover, many people did not want to visit the dental studios due to the fear of virus transmission. Moreover, as proved in many studies in pre-pandemic time, the prolonged lack of regular oral health check-ups may lead to periodontitis [9,10]. Another unprecedented situation happened during the COVID-19 pandemic. Greater demand for personal protective equipment (PPE) was detected globally and led to a shortage in masks, gloves, and almost all PPE [11]. Difficult access to PPE was significant and obvious in the early phase of the COVID-19 pandemic. In order to minimize the viral spread risk during the current COVID-19 pandemic and post-pandemic times, the global project to define the best organization of dental offices was conducted [12].

Rapid changes in the epidemiological situation might be easily and strongly visible in the surveillance of the more widely used search engines, for example, the Google search engine, the most used engine with the vast majority of the market share, as much as 92.26% [13].

The aim of this study was to analyze the impact of the COVID-19 pandemic on oral health and the interest in teledentistry.

## 2. Materials and Methods

The data for the study were collected from the open database—Google Trends (GT) service among anonymous search engine users related to oral health and teledentistry subjects [14,15]. Each data record represents a weekly worldwide number of queries processed by Google engine and available according to the selected time period. In the study, the analyzed material was collected from 19 June 2016 to 6 June 2021. All data imported from GT represent queries were normalized to the time and location. The resulting numbers are scaled on a range of 0 to 100, where 100 is the maximum number of searches in a stated period of time. The following expressions were analyzed: “dental care”, “emergency dental care”, “oral health”, “periodontitis”, “COVID-19” and ”PPE dentist”, as well as the phrases “teledentistry” and “is it safe to go to the dentist”, were investigated to check the interest for online consultations.

The data analysis was divided into two sections. The first section provides the detailed level description of data, its structure, and main samples characteristics needed in order to perform further calculations. In the second section, the pairwise correlation between data series was analyzed. For each time series data, the trend, cyclical, and random fluctuations analyses were performed. The purposes of this section were as follows:

- To present pairwise time series visualizations;
- To perform time series decompositions indicating the trend, cyclical, and random fluctuations;
- To analyze the cross-correlation between chosen time series pairs with statistically significant estimation;
- To evaluate a pairwise of mutual influence (where possible).

To estimate the dynamics rate (hereinafter), the comparisons of two mean rates for two periods were made: first one for the period before the 22–23 January 2020, when the World Health Organization (WHO) Director gathered an Emergency Committee to discuss whether the new virus outbreak determined a public health emergency of worldwide concern [16] (that refers to dates from 19 June 2016 to 19 January 2020 in our weekly split data set) and the second one from 19 January 2020 to 6 June 2021. For simplicity, we further refer to those periods as before and during the pandemic. In the study, we aimed to check the time before the official pandemic onset, as the search engines may have shown some interest in the subject before official regulations were made.

For the qualitative and quantitative analysis of the correlation, the time series collections were divided into the following 4 pairs:

- “Dental care”–“emergency dental”;
- “Oral health”–“periodontitis”;
- “Teledentistry”–“is it safe to go to the dentist”;
- “COVID-19”–“PPE-dentist”.

All statistical estimations with data visualizations were programmed by the “R” programming language [17] with “R studio” version 1.4.1106 open-source software for data science, scientific research, and technical communication [18].

## 3. Results

### 3.1. Time Series Visualizations

A graphical illustration of time series was presented by a paired graph of the dynamics of requests in the study period. For the purposes of analyzing the trend, cyclical, and random fluctuations, the time series were decomposed for each collection separately.

**Figure 1:**
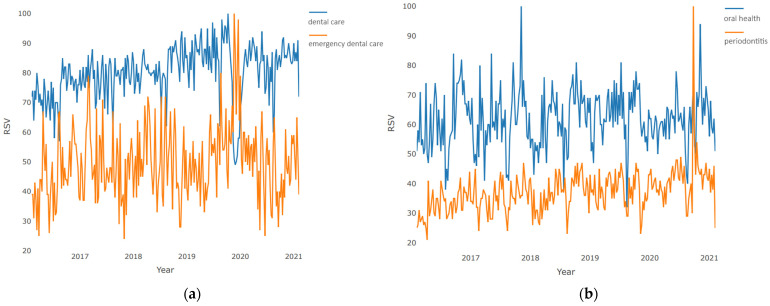
Pairwise comparison of weekly requests during 2016–2021: (**a**) “dental care”–“emergency dental care”; (**b**) “oral health”–“periodontitis”.

The pairwise time series visualization of “dental care”–“emergency dental care” is presented in Figure 1a. The plot clearly shows a high proportion of uncertainty in demand for dental services at the very beginning of 2020. This is evidenced by a sharp decline in demand for planned dental procedures with a simultaneous sharp increase in demand for emergency ones. However, this phase had a short-term character, and during the next 1–2 months, the structure of demand returned to its normal state.

The pairwise, time series visualization of “oral health”–“periodontitis” is presented in Figure 1b. The plot shows large interest growth when regarding “periodontitis” with the peak at the beginning of the year 2020 with stable and slight growth in the number of questions asked during the analyzed period. The number of queries for “oral health” is more stable. A peak in “periodontitis” queries was detected after the hard lockdown and the lower interest in the “oral health” subject.

**Figure 2:**
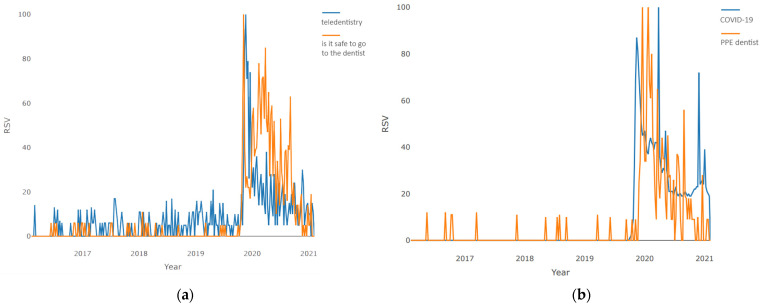
Pairwise comparison of weekly requests during 2016–2021: (**a**) “teledentistry”–“is it safe to go to the dentist”; (**b**) “COVID-19”–“PPE dentist”.

Furthermore, the pairwise, time series visualization of “teledentistry”–“is it safe to go to the dentist” is presented in Figure 2a. Both terms under consideration rarely occurred in the pre-pandemic time. Only at the beginning of 2020 did these concepts become widespread. The plot clearly shows a peak when the request for both terms reaches its maximum. The demand for the “teledentistry” term then begins to decline exponentially until the end of the period under review, reaching levels preceding the pandemic, and the expression “is it safe to go to the dentist” is characterized by steady demand throughout almost all of 2020, reaching local highs during periods of lockdowns and exacerbations of restrictions. However, at the end of the period under review, requests for this term also reached a minimum, slightly exceeding pre-pandemic levels.

The pairwise, time series visualization of “COVID-19”–“PPE dentist” is presented in Figure 2b. Both expressions came into use immediately with the onset of the pandemic. The demand curve for “COVID-19” is characterized by the presence of three global highs that occurred at the junction of 2019–2020, in the first quarter of 2020, and at the junction of 2020/2021, which corresponds to the time of global pandemic waves. The topic “PPE dentist” standing for personal protective equipment was characterized by the greatest demand at the beginning of the pandemic; over the next 1.5 years, the level of interest dropped exponentially, until the end of the period under review, where it again reached the pre-pandemic level.

### 3.2. Trends Analysis

**Figure 3:**
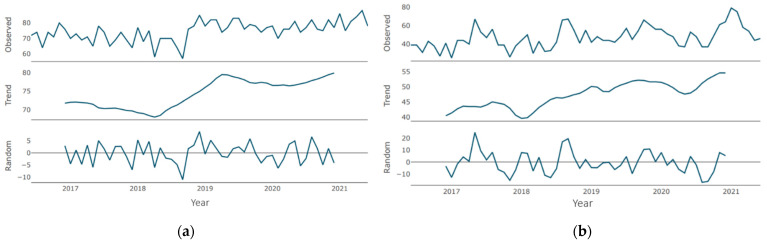
Decomposition of time series (month granularity): (**a**) “dental care”; (**b**) “emergency dental care”.

Regarding the “dental care” time series, during the period under consideration, there are two periods of trend growth Figure 3a: the first (more pronounced) from the beginning of 2018 to the beginning of 2019, and the second (more smoothed) from the beginning of 2020. There was no downtrend during the pandemic period. Regarding the “emergency dental care” time series (Figure 3b), the direction is multidirectional (with local ups and downs) with a general upward trend. After reaching the minimum in 2018 through mid-2020, there was a significant increase, followed by a short-term moderate decline (probably during the general lockdown). An upward trend was observed since the second half of 2020, which reached its maximum values at the end of the period under review.

**Figure 4:**
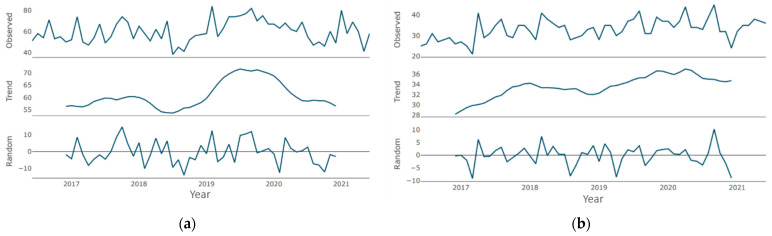
Decomposition of time series (month granularity): (**a**) “oral health”; (**b**) “periodontitis”.

Regarding the “oral health” time series, during the period under consideration, there are two periods of trend growth Figure 4a: the first (minor one) from the beginning of 2017 to the beginning of 2018, and the second (a pronounced one) from mid-2018 to early 2020. Periods of growth are followed by periods of decline with similar severity and duration. Since the beginning of the pandemic, a negative trend was recorded with small periods of stabilization with a tendency to smooth out. There were no upward trends. In mid-2021, the level of demand is close to the minimum values for the period under review. Furthermore, regarding the “periodontitis” time series (Figure 4b), for most of the period under review, the trend was characterized as moderately growing with small corrections and short declines. Since the beginning of 2019, there has been protracted growth, which reached its maximum during the pandemic. At the end of the period under review, there is a slight decrease in the trend, which, however, exceeds the demand levels of the pre-pandemic period.

**Figure 5:**
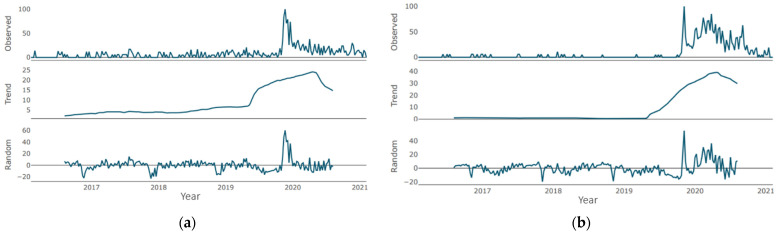
Decomposition of time series (week granularity): (**a**) for “teledentistry”; (**b**) for “is it safe to go to the dentist”.

Regarding the “teledentistry” time series (Figure 5a), until the second half of 2019, the trend was an almost flat curve, with a barely noticeable increase, which is replaced by a sharp growth phase lasting about a year. The growth for the specified period was about 20 points. Then, the growth phase was replaced by a moderate decline phase, which continued until the end of the period under review. Similarly, in the “is it safe to go to the dentist” time series (Figure 5b), until the second half of 2019, the trend was a flat line with a zero value, which was replaced by a phase of sharp growth that lasted for about a year. The growth for the indicated period was about 40 points. Then, the growth phase gave way to a moderate decline phase, which lasted until the end of the period under review.

**Figure 6:**
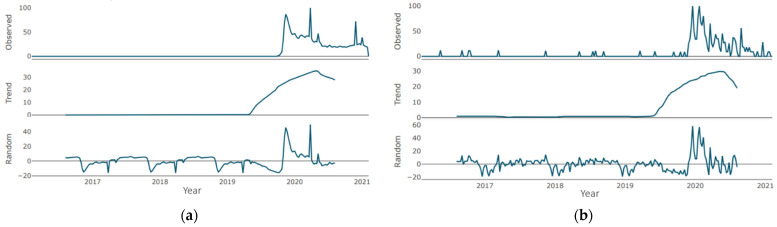
Decomposition of time series (week granularity): (**a**) for “COVID-19”; (**b**) for “dentist PPE”.

The trend line for both expressions in Figure 6a,b—“COVID-19” and “PPE dentist”—repeats the pattern of the previous pair. Until mid-2019, a horizontal line at the 0 level is observed, after which the annual growth phase with a maximum increase of up to 30 points is observed. The growth phase is replaced by a moderate decline phase, which continues until the end of the period under consideration (the volume of decline for “COVID-19”—5 points, for “PPE dentist”—10 points).

### 3.3. Cyclical and Random Fluctuations Indications Analysis

In the “dental care” time series (Figure 3a), there is a single negative peak of average severity in the second half of 2018. There were no positive peaks in the similar intensity. When regarding the “emergency dental care” time series Figure 3b, there are two peaks: the most pronounced at the beginning of the period (breaking through the 20 marks) and slightly less pronounced in the second half of 2018 (approaching the 20 marks). Negative peaks of similar intensity were not observed.

Regarding the “oral health” time series (Figure 4a), there are several cycles of low severity breaking through the 10-point mark in both directions. Moreover, in the “periodontitis” time series (Figure 4b), there are several peaks of average severity approaching the 10-point mark in both directions. The most pronounced is the positive cycle at the end of the period under consideration.

In the “teledentistry” time series (Figure 5a), there is one pronounced cycle with an increase in the parameter of about 60 points, and there is one pronounced cycle with an increase in the parameter of more than 40 points when describing the “is it safe to go to the dentist” time series (Figure 5b).

Two pronounced cycles are clearly visible in the “COVID-19” time series in Figure 6a—at the junction of 2019/2020 and in the first half of 2020, with an increase of about 35 points. No pronounced negative peaks are observed. A series of peaks regarding the expression “PPE dentist” at the junction of 2019/2020 with gains of more than 50 points is clearly visible. No pronounced negative peaks were recorded (Figure 6b).

### 3.4. Changes in Request Average during COVID-19 Pandemic

The mean of “dental care” time series request rate in the pre-pandemic period has a rate of 79.9; during the pandemic, the mean rate was increased to 80.2. Thus, from the pandemic onset to the present, the “dental care” requests mean rate has remained particularly unchanged (the increasing rate is about 0.4%). Moreover, the mean of the “emergency dental care” time series request rate in the pre-pandemic period has a rate of 48.73, and during the pandemic, the mean rate increased to 52.6. Thus, from the pandemic onset to the present, the main rate of “emergency dental care” increased by 7.9%. The mean of the “oral health” time series request rate in the pre-pandemic period has a rate of 62.0; during the pandemic, the mean rate changed to 61.8. Thus, from the pandemic onset to the present, the “oral health” requests mean rate remained particularly unchanged (the decreasing rate is about 0.3%). The mean of the “periodontitis” request rate in the pre-pandemic period has a rate of 35.7; during the pandemic, the mean rate changed to 41.1. Thus, from the pandemic onset to the present, the main rate of “periodontitis” increased by 15.1%. Moreover, the mean of the “teledentistry” time series request rate in the pre-pandemic period has a rate of 4.3; during the pandemic, the mean rate changed to 21.4. Thus, from the pandemic onset to the present, the “teledentistry” requests mean rate characterized by a 5 times increase. The mean of the request rate for the “is it safe to go to the dentist” time series in the pre-pandemic period has a rate of 0.8; during the pandemic, the mean rate changed to 31.9. Thus, from the pandemic onset to the present, the requests mean rate is characterized by an increase of almost 40 times. The mean of the “PPE dentist” request rate in the pre-pandemic period has a rate of 0.8; during the pandemic, the mean rate changed to 24.0. Thus, from the pandemic onset to the present, the “PPE dentist” request mean rate is characterized by an increase of 30 times. When regarding the “COVID-19” time series, this expression did not exist before the pandemic.

### 3.5. Cross-Correlation

**Figure 7:**
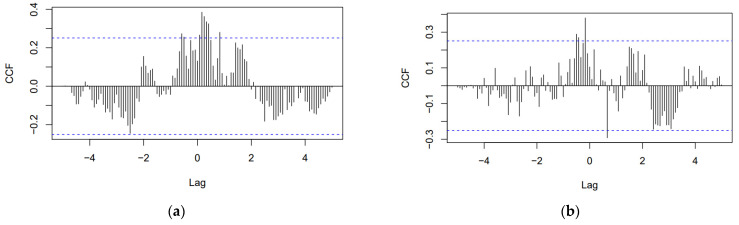

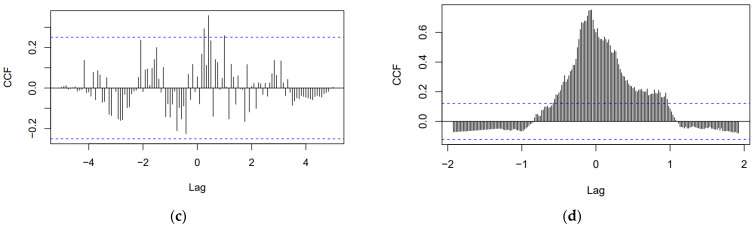
Cross-correlation plots: (**a**) “dental care”–“emergency dental” (granularity: month); (**b**) “oral health”–“periodontitis” (granularity: month); (**c**) “teledentistry”–“is it safe to go to the dentist” (granularity: month); (**d**) “COVID-19”–“PPE dentist” (granularity: week).

The time series pair “dental care”–“emergency dental” cross-correlation function plot is shown in Figure 7a. There are a number of significant correlations on both sides of 0. All significant correlations are positive, from which it follows, an increase in one parameter leads to an increase in another. There is no significant correlation at h = 0. Furthermore, the time series pair “oral health”–“periodontitis” cross-correlation function plot is shown in Figure 7b. The correlation magnitude and the number of significant correlations are greater on the left side of 0. The most dominant cross-correlation occurs at h = −2. The correlation is positive, indicating that an above-average value of “oral health” is likely to lead to an above-average value of “periodontitis” about 2 months later. Additionally, a below-average value of “oral health” is associated with a likely below-average “periodontitis” value about 2 months later. Moreover, the time series pair “teledentistry”–“is it safe to go to the dentist” cross-correlation function plot is shown in Figure 7c. There are a number of significant correlations only on the right side of 0. All significant correlations are positive. Finally, the time series pair “COVID-19”–“PPE dentist” cross-correlation function plot is shown in Figure 7d. There are a number of significant correlations on both sides of 0. There is also a significant correlation at h = 0. All significant correlations are positive, from which it follows, an increase in one parameter leads to an increase in another. An above-average value of “COVID-19” is highly likely to lead to an above-average value of “PPE dentist” about 4 weeks later. Since both expressions existed in GT searches for less than 2 years, the use of month granularity was impossible, which is why, in this case, a week granularity was used.

## 4. Discussion

The aim of the study was to investigate the impact of the COVID-19 pandemic on oral health and teledentistry subjects. To the best of our knowledge, this is one of the few studies [19,20] tracing the GT expressions connected with oral health during the COVID-19 pandemic. The first noticeable outcome of the research was a large increase in emergency dental care questions asked during the spring lockdown in 2020 with a simultaneous decrease in regular dental care queries. Our results strongly show that the COVID-19 pandemic had significantly influenced dental-care-seeking behavior. According to the recommendations and because of the fear surrounding the epidemic, people were averse to be outside and less willing to visit dental studios. Moreover, dental care was not widely available during the early phase of the pandemic, with a large number of patients expected to seek emergency dental service only when urgent care was needed [5]. In line with our study, the outcomes of the study conducted by Faccini et al. indicate that during the hard lockdown, 64.6% of dentists carried out only emergency treatments, with 26.1% of dentists still maintaining the routine planned appointments, and with 9.3% of dental offices closed for the duration of the lockdown. An increase in urgent dental procedures was noted by 44.1% of the dentists. It occurred most often due to the lower availability for a longer period of time for regular dental care. The main causes of emergency appointments were toothache, dental trauma, or broken restorations [21]. Similarly, a study conducted in Beijing, China, with 2537 patients involved in the study, indicated that at the beginning of the COVID-19 pandemic, 38% fewer patients sought dental emergency care than before. Dental problems and oral infection increased from 51.0% in the pre-pandemic period to 71.9% during the COVID-19 pandemic period [5]. A finding in line with our study is that the COVID-19 pandemic outbreak had strongly influenced the emergency dental services. The large influence of COVID-19 disease on oral cavity was shown in the study conducted by Gherlone et al., as 83.6% of patients reported anomalies of the oral cavity, such as dry mouth or salivary gland ectasia, that lasted even up to 3 months after hospitalization due to COVID-19 [22].

According to recent studies, people with serious medical conditions such as diabetes, heart diseases, lung or kidney chronic disease, and older persons were at high risk for developing more severe COVID-19 infection. On the other hand, poor oral health and hygiene may increase the risk of developing the abovementioned medical problems. Therefore, improving oral health among the whole population may lead to a lower risk of developing systemic diseases and, in this way, reduce the morbidity due to COVID-19 [9,23]. In the study among a group of 568 patients, the higher risk of intensive care unit admission with the need for assisted ventilation and worse disease outcomes, leading even to the death of COVID-19 patients, was associated with periodontitis. Similarly, COVID-19 patients with periodontitis had significantly higher blood levels of D-dimer and C-reactive protein and white blood cells [24]. After a lower level of interest in oral health was detected in the number of queries asked in GT during the hard lockdown, in the conducted study, we detected a peak in periodontitis questions and about a 15% interest growth in the subject.

During the pandemic, telemedicine represented an opportunity to improve accessibility to medical treatments. According to some studies, teledentistry could be comparable to face-to-face visits for oral screening, especially in areas with limited access to oral health care. In fact, the identification of oral pathologies via teleconsultations is widely possible and valid [25]. The online examination using intraoral scans may become helpful in detecting dental problems. The remote assessment of intraoral scans can allow an efficient screening and the correct triage of patients. Improved intraoral scans can provide even three-dimensional images with true colors [26]. Nowadays, to minimize contact with the patient and to ensure patient safety clinicians may follow up with patients online, for example, through video calls. In the present study, a 5-time increase in query rates for teledentistry during the COVID-19 pandemic was detected because of the anxiety to visit the dentist. The query “is it safe to go to the dentist” increased the question rate during the pandemic almost 40 times. It may indicate the usefulness of such a digital tool during epidemiological threats. The screening for other chronic viral infections was previously conducted in dental offices, for example, for HCV virus [27]; such easy, free-of-charge prevention campaigns may use teleconsultation in the post-pandemic time.

In addition, specific recommendations and lots of preventive measures against COVID-19 were undertaken in dental offices with infection control strategies. The urgent need for dental emergency care required fast delivery of the appropriate PPE. The initial shortage and distribution challenges of PPE was seen globally [11,28], as well as the large interest in PPE connected with dentists was observed in our study, with a 30-time increase of requests mean rate. The patient management protocols were applied to reduce the risk of infection and to prevent the spread of the cross-infection, for example, clinical triage for patient screening with a questionnaire on recent disease symptoms, body temperature measurement, avoiding crowding in the waiting room, a hand sanitizer available to patients before entering the operating rooms, and the use of PPEs and oral rinses with hydrogen peroxide before the dental treatment [29,30,31,32]. The WHO recommended the use of FFP3 masks according to the European terminology or N100, according to the United States nomenclature [33]. Members of the oral healthcare team should be acquainted with the COVID-19 transmission and preventive measures, as the oral cavity is an important site for COVID-19 infection and a potential route of virus transmission [34]. Cross-infection control measures should be applied to all patients because asymptomatic COVID-19 persons may also need emergency dental treatment [35].

This study has some limitations. Firstly, the analyzed data were collected from one search engine; nevertheless, Google engine has nearly four billion users worldwide and covers more than 90% of all queries on the internet, with near 7 billion queries per day. With 4.39 billion internet users worldwide, the number of Google users is globally nearly four billion [36]. In our study, the GT request sample size for 5 years period is about big data collections. Thus, the nature of the data and sample size is appropriate for the purpose and nature of the study. Moreover, when considering the overlapping of expressions meaning, in a small sample, it might be discussed as a limitation; however, the sample of expressions gathered in the presented study is huge, the time of observation is long, and therefore, it should not induce significant changes in results. Secondly, the study was carried out globally, in order not to add too much manual categorization, by providing expressions in different languages. When adding queries in many languages across countries, it becomes more difficult to choose the best fitting expression used in a specific country. Finally, there are no strict rules on how to analyze the GT in health care research [37]; on the other hand, the GT data are recognized by the scientific community as a reliable source on the basis of which scientific papers in various fields are published [38,39]. Because of the anonymized approach of data collection, GT enables the analysis and forecasting of sensitive topics especially in medicine [37,40,41,42,43]. Because of using the revealed and not stated users’ preferences, it is possible to obtain information that would be otherwise impossible to collect. Moreover, GT offers a substantial promise for the global monitoring of diseases in countries that lack clinical surveillance but have sufficient internet coverage to allow for surveillance via digital epidemiology [44].

## 5. Conclusions

1. Firstly, according to the search queries analysis from GT, the COVID-19 pandemic had a large impact on oral health problems;
2. Moreover, emergency dental care became more required during the onset of pandemic and hard lockdown than regular dental care;
3. Finally, teledentistry gained in popularity during the lockdowns according to globally asked questions in GT service.

## Data Availability

The data presented in this study are available on request from the corresponding author.
